# Association of blood-activating commercial Chinese polyherbal preparation with clinical outcomes in older patients with ischemic cardiovascular or cerebrovascular diseases: a real-world cohort study

**DOI:** 10.3389/fphar.2026.1819226

**Published:** 2026-06-17

**Authors:** Su Su, Xianzhe Dong, Moyi Wang, Beibei Li, Xiaoxuan Xing, Pei Gao, Lan Zhang

**Affiliations:** 1 Department of Pharmacy, Xuanwu Hospital, Capital Medical University, Beijing, China; 2 Institute of Advanced Clinical Medicine, Peking University, Beijing, China; 3 Department of Epidemiology and Biostatistics, Peking University, Beijing, China; 4 Key Laboratory of Epidemiology of Major Diseases, Peking University, Beijing, China

**Keywords:** activating blood and resolving stasis, elderly patients, ischemic cardiovascular disease, ischemic cerebrovascular diseases, secondary prevention

## Abstract

**Background:**

Commercial Chinese polyherbal preparation for activating blood and resolving stasis (CPABRS) were widely used in combination with guideline-directed antiplatelet agents (AA). However, real-world evidence on the effectiveness and safety of this combination remains limited, particularly among older patients with ischemic cardiovascular and cerebrovascular diseases (ICCD).

**Method:**

This retrospective cohort study used data from the Beijing Municipal Medical Insurance Database (BMMID). Patients aged ≥65 years with ICCD were categorized into three groups: AA + CPABRS, CPABRS alone, and AA alone. Logistic regression was used to estimate odds ratios (ORs) and 95% confidence intervals (CIs) for major adverse cardiac and cerebrovascular events (MACCEs) and bleeding outcomes. Cox proportional hazards regression, inverse probability of treatment weighting (IPTW) based on propensity scores, subgroup and sensitivity analyses were performed to examine the robustness of the findings.

**Results:**

A total of 12,113 patients were included (median age, 77 years); 78.2% received AA + CPABRS, 11.5% received CPABRS alone, and 10.4% received AA alone. Compared with AA alone, AA + CPABRS was associated with a lower risk of MACCEs (OR 0.687, 95% CI 0.544–0.876) without a statistically increase in the risk of bleeding (OR 0.734, 95% CI 0.417–1.399). CPABRS monotherapy was associated with a non-significant reduction in MACCEs (OR 0.820, 95% CI 0.597–1.126) and a numerically higher but imprecise bleeding risk (OR 1.831, 95% CI 0.895–3.866). The Cox, IPTW-propensity scores analysis, subgroup and sensitivity analyses yielded consistent results.

**Conclusion:**

In older ICCD patients, adjunctive CPABRS combined with antiplatelet therapy was associated with lower risk of MACCEs without statistically significant increase in bleeding events during short-term follow-up. These findings suggest that CPABRS may be a potentially useful complementary strategy for secondary prevention in this population.

## Introduction

Ischemic cardiovascular and cerebrovascular diseases (ICCD), such as myocardial infarction and ischemic stroke, are major causes of morbidity and mortality in older adults and impose a substantial socioeconomic burden worldwide (Vaduganathan et al., 2022). Although guideline-directed antiplatelet agents (AA) remain the cornerstone of secondary prevention in older patients with ICCD (Antithrombotic Trialists' Collaboration, 2002; Kang et al., 2023), these patients continue to be at high risk for both major adverse cardiac and cerebrovascular events (MACCEs) and bleeding complications (Cutlip et al., 2015; Généreux et al., 2015; Valle et al., 2016). Therefore, there is an urgent need to identify effective and safe adjunctive therapeutic strategies in this high-risk population.

In Traditional Chinese Medicine (TCM), ICCD falls under the category of Blood Stasis Syndrome, a pathological condition marked by impaired blood circulation, microvascular obstruction, abnormal hemorheology and hemodynamics, and ischemia of affected tissues (Chen, 2012). Accordingly, the core therapeutic principle is to “promoting blood circulation and removing blood stasis”. Commercial Chinese polyherbal preparation for activating blood and resolving stasis (CPABRS) aim to improve blood flow, enhance microcirculation, and alleviate ischemic injury. In China, CPABRS have been widely utilized in the management of ischemic disorders in contemporary clinical practice. Our previous study found that among older ICCD patients, 78.1% received CPABRS plus antiplatelets (AA), and 11.5% received CPABRS alone for secondary prevention. However, only a few CPABRS (e.g., Tongxinluo, Xinyue, Shexiang Baoxin) have shown reduced MACCEs and bleeding risks in RCTs (Ge et al., 2021; Guo et al., 2020; Yang et al., 2023). By contrast, other CPABRS (e.g., Ginkgo, Xuesaitong, Danqi, Sanchitongshu, Danhong), have mainly been reported to improve neurological function, angina severity, and functional scores (Chen et al., 2009; Chen et al., 2022; He et al., 2011; Wu et al., 2023; Zhang et al., 2023). Existing studies largely focused on symptom relief, not specifically targeted on MACCEs. Despite the widespread use of CPABRS, real-world evidence on whether CPABRS (alone or with AA) lowers MACCEs and bleeding in older ICCD patients remains limited.

To address this gap, we conducted a population-based real-world study using data from the Beijing Municipal Medical Insurance Database (BMMID) to evaluate the clinical outcomes (rehospitalization for MACCEs) and safety (rehospitalization or outpatient visits for bleeding events) of CPABRS, alone or in combination with AAs, in old Chinese patients with ICCD. Because all CPABRS share the same therapeutic principle of activating blood and resolving stasis and are often used interchangeably in clinical practice, they were analyzed as a single therapeutic class in this study. This study adds population-based real-world evidence on CPABRS and may help clarify the potential role of CPABRS in secondary prevention for ICCD in older adults.

## Methods

### Study design and data source

This retrospective population-based cohort study was conducted using data from BMMID, a comprehensive medical claims database administered by the Beijing Municipal Medical Insurance Bureau (Su et al., 2022; Su et al., 2025). The BMMID captures approximately 95% of healthcare utilization among Beijing residents and includes detailed information on outpatient visits, outpatient prescriptions, and hospitalizations.

### Study population

Individuals aged ≥65 years who were discharged with a discharge diagnosis of ICCD were initially identified. Eligible diagnoses included myocardial infarction, unstable angina, ischemic stroke, percutaneous coronary intervention (PCI), and coronary artery bypass grafting. Detailed definitions of eligible diagnoses are provided in Supplementary Appendix 1. Participants were excluded if they 1) had a diagnosis of cerebral hemorrhage on or before the index date, 2) had no record of antiplatelet therapy or CPABRS use during the observation period, or 3) had a baseline date that coincided with the end of follow-up.

### Index date and follow-up

The index date was defined as the date of first discharge with a diagnosis of ICCD. Follow-up began on the index date, when patients initiated CPABRS and/or antiplatelet therapy, and continued until the first occurrence of a study outcome or the end of the corresponding observation period (30 September 2016, or 30 September 2017), whichever occurred first. Participants who did not experience any study outcome were censored at the end of the follow-up. Follow-up time was calculated as the interval from the index date to the date of outcome event or censoring.

### Definition of ischemic cardiovascular and cerebrovascular diseases

ICCD was identified using discharge diagnoses recorded in the hospitalization dataset. Because ICD-10 coding in the BMMID was sometimes incomplete, the study population was identified using both diagnostic text and ICD-10 codes. The following ICD-10 codes were applied: myocardial infarction: I21–I25; ischemic stroke: I63–I64, I69; unstable angina: I20; PCI: procedure codes 36.06–36.07; and coronary artery bypass grafting: 36.10–36.14. The Chinese diagnostic terms used to identify the study population are provided in Supplementary Appendix 1.

### Definition of CPABRS and antiplatelet agents

Commercial Chinese polyherbal preparations for activating blood and resolving stasis (CPABRS) were defined according to Contemporary Clinical Pharmacology (Chen et al., 2018) and corresponding package inserts. Given their shared therapeutic principle of activating blood and resolving stasis and their interchangeable use in routine clinical practice, these products were analyzed as a single therapeutic class. Detailed information on the composition, indications, adverse reactions, and potential drug interactions of each CPABRS product is provided in Supplementary Appendix 1.

Antiplatelet therapy (AA) was defined in accordance with current guideline recommendations. For ischemic cardiovascular disease, AA comprised dual antiplatelet therapy with aspirin plus clopidogrel or aspirin plus ticagrelor (Rao et al., 2025). For ischemic cerebrovascular disease, AA comprised antiplatelet monotherapy with aspirin or clopidogrel (Kleindorfer et al., 2021).

Therapy initiation was defined as the first prescription recorded after index diagnosis. Participants were classified into three groups based on treatment exposure after the index date: 1) AA + CPABRS group, defined as any record of CPABRS use in combination with AA; 2) CPABRS group, defined as CPABRS without AA; and 3) AA group, defined as AA use without CPABRS.

Treatment group assignment was based on prescriptions recorded between baseline and the end of follow-up, regardless of subsequent changes in medication use.

### Outcome measures

#### Primary outcome

The primary endpoint was rehospitalization for MACCEs, defined as a composite of myocardial infarction (I21–I25), unstable angina pectoris (I20), percutaneous coronary intervention (PCI; 36.06–36.07), coronary artery bypass grafting (36.10–36.14), and ischemic stroke (I63–I64).

Outcome events were identified during follow-up by linking each participant’s unique patient identification number to hospitalization records in the database. Participants were considered to have reached the primary endpoint if any of these events occurred after the index date. Detailed diagnostic descriptions and coding rules are provided in Supplementary Appendix 1.

#### Secondary outcome

The secondary endpoint was rehospitalization or outpatient visits for bleeding events, defined in accordance with the International Society on Thrombosis and Hemostasis (ISTH) criteria (Kaatz et al., 2015; Schulman et al., 2005). The composite bleeding events included intracranial hemorrhage (I61–I62), gastrointestinal bleeding (K25, K26, K27, and K92), intraocular bleeding (H35), gingival bleeding (K06.802), and recurrent or persistent hematuria (N02).

Secondary outcome events were identified during follow-up by linking each patient’s identification numbers to outpatient and hospitalization records. Participants were considered to reach the secondary endpoint if at least one record met the predefined bleeding criteria after the index date. Detailed outcome definitions, diagnostic descriptions, and coding procedures are provided in Supplementary Appendix 1.

### Covariates

Baseline covariates were selected based on the known risk factors for MACCEs and bleeding. Demographic variables included age (continuous) and sex (female as the reference). Comorbidities (binary variables with no as the reference) included hypertension, diabetes mellitus, hyperlipidemia, other cerebrovascular diseases, chronic kidney disease, gastroduodenal ulcers, embolic diseases, and aortic dissection or aneurysms. Concomitant medications (binary with no as the reference) included antihyperlipidemic drugs, antihypertensive agents, hypoglycemic drugs, and anticoagulants.

### Statistical analysis

The baseline characteristics were summarized using descriptive statistics. Continuous variables are reported as medians with interquartile ranges (IQR), and categorical variables as counts and percentages. Logistic regression models were used to assess the association of AA + CPABRS and CPABRS monotherapy with clinical outcomes, with the AA group as the reference. Adjusted odds ratios (ORs) with 95% confidence intervals (CIs) were estimated for both the primary and secondary outcomes. In addition to logistic regression, Cox proportional hazards regression was performed as a time-to-event analysis to further assess the robustness of the associations between exposures and outcomes. To further reduce treatment-selection bias arising from baseline group imbalance, inverse probability of treatment weighting (IPTW) based on propensity scores was applied. Missing data were handled using worst-case imputation. All statistical analyses were conducted using the R software (version 4.5.0).

### Subgroup analyses

Subgroup analyses were performed to explore potential effect modifications. The predefined subgroups included sex, age (≤75 or >75 years), comorbid hypertension, diabetes mellitus, and specific CPABRS types. In addition, the most frequently prescribed CPABRS, including Yinxing (Ginkgo biloba), Danshen (Salvia miltiorrhiza Bunge), Danhong (Salvia miltiorrhiza; Carthamus tinctorius), Sanqi (Panax notoginseng), and Shuxuetong (Hirudo; *Eisenia fetida*), were examined separately. The CPABRS were validated taxonomically, and the full species names including authorities, family and drug name were provided in Supplementary Appendix 1.

### Sensitivity analyses

Two sensitivity analyses were conducted to assess the robustness of the findings. First, participants with baseline gastroduodenal ulcers were excluded. Second, participants with baseline ischemic cardiovascular disease were excluded.

### Ethics approval

This study was approved by the Ethical Review Board of Xuanwu Hospital, Capital Medical University (Clinical Scientific Research [2018] no. 24, approval date 25 June 2018).

## Results

### Study population and baseline characteristics

**FIGURE 1:**
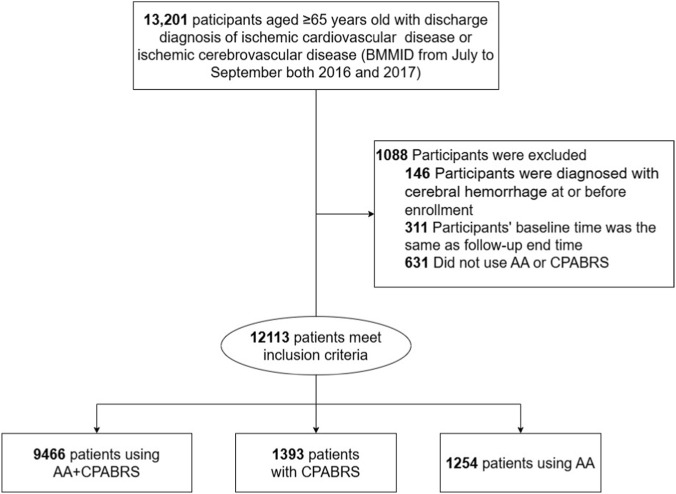
Flowchart of participant selection. Abbreviations: BMMID: Beijing Municipal Medical Insurance Database; AA: antiplatelet agents; CPABRS: Chinese patent medicine for activating blood and resolving stasis.

A total of 13,201 individuals aged ≥65 years with ischemic cardiovascular or cerebrovascular diseases were identified in the BMMID between July and September 2016 and 2017. After excluding patients with a history of cerebral hemorrhage (n = 146), those without any record of antiplatelet therapy or CPABRS use (n = 631), and those whose baseline data coincided with the end of the follow-up (n = 311), 12,113 patients were included in the final analytic cohort (Figure 1).

Of these patients, 9,466 (78.2%) patients were classified as receiving AA + CPABRS, 1,393 (11.5%) as receiving CPABRS alone, and 1,254 (10.4%) as receiving AA alone. The total follow-up duration for the study population ranged from 1 to 91 days. The median follow-up duration was 43 days (IQR, 19–66) in the AA + CPABRS group, 35 days (IQR, 15–61) in the CPABRS group, and 36 days (IQR, 12–60) in the AA group.

The baseline characteristics are presented in Table 1. The median age differed across the three groups (77, 79, and 76 years for AA + CPABRS, CPABRS, and AA users, respectively; p < 0.001). The proportion of female patients was 40.5% among AA + CPABRS users, 51.9% among CPABRS users, and 41.9% among AA users.

**TABLE 1:** Patient demographic and baseline characteristics.

Variable	AA + CPABRS (n = 9466)	CPABRS (n = 1,393)	AA (n = 1,254)	Total (n = 12,113)	p
Age, median (Q1, Q3)	77.0 (70.0–82.0)	79.0 (72.0–83.0)	76.0 (70.0–81.0)	77.0 (71.0–82.0)	<0.001
Female, n (%)	3,831 (40.5%)	723 (51.9%)	526 (41.9%)	5,080 (41.9%)	<0.001
Antihyperlipidemic drugs, n (%)	8,431 (89.1%)	744 (53.4%)	1,045 (83.3%)	10,220 (84.4%)	<0.001
Antihypertensive drugs, n (%)	8,071 (85.3%)	1,065 (76.5%)	1,047 (83.5%)	10,183 (84.1%)	<0.001
Hypoglycemic drugs, n (%)	4,382 (46.3%)	501 (36.0%)	595 (47.4%)	5,478 (45.2%)	<0.001
Anticoagulants, n (%)	3,656 (38.6%)	603 (43.3%)	607 (48.4%)	4,866 (40.2%)	<0.001
Hypertension, n (%)	2,499 (26.4%)	442 (31.7%)	502 (40.0%)	3,443 (28.4%)	<0.001
Diabetes mellitus, n (%)	1,228 (13.0%)	224 (16.1%)	276 (22.0%)	1728 (14.3%)	<0.001
Hyperlipidemia, n (%)	1,234 (13.0%)	124 (8.9%)	204 (16.3%)	1,562 (12.9%)	<0.001
Other cerebrovascular disease, n (%)	213 (2.25%)	14 (1.01%)	42 (3.35%)	269 (2.22%)	<0.001
Chronic kidney disease, n (%)	153 (1.62%)	36 (2.58%)	39 (3.11%)	228 (1.88%)	<0.001
Gastroduodenal ulcers, n (%)	30 (0.32%)	7 (0.50%)	5 (0.40%)	42 (0.35%)	0.517
Embolic disease, n (%)	20 (0.21%)	9 (0.65%)	6 (0.48%)	35 (0.29%)	0.008
Aortic dissection or aneurysm, n (%)	13 (0.14%)	0 (0.00%)	7 (0.56%)	20 (0.17%)	0.001

AA, antiplatelet agents; CPABRS; commercial Chinese polyherbal preparation for activating blood and resolving stasis; ICCD, Ischemic cardiovascular or cerebrovascular diseases.

Other cerebrovascular diseases included carotid artery stenosis, cerebral artery stenosis, and vertebral artery stenosis.

Embolic disease included peripheral arterial occlusion and pulmonary embolism.

Significant differences were also observed in comorbidities and concomitant medications. Compared to the other groups, AA users exhibited a higher prevalence of hypertension (40.0%), diabetes mellitus (22.0%), hyperlipidemia (16.3%), chronic kidney disease (3.11%), aortic dissection/aneurysm (0.56%), and other cerebrovascular diseases (3.35%). AA users also more frequently received hypoglycemic drugs (47.4%) and anticoagulants (48.4%).

### Primary outcome: MACCEs

#### Incidence of MACCEs

During the follow-up period, 658 patients (5.4%) experienced MACCEs. The incidence of MACCEs was 5.09%, 6.32%, and 7.02% in the AA + CPABRS, CPABRS, and AA groups, respectively (p = 0.006).

#### Association between treatment and MACCE risk

**FIGURE 2:**
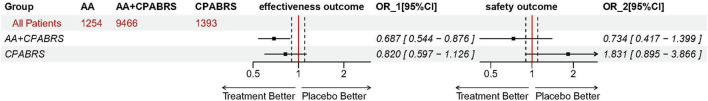
Association of treatment group with primary (effectiveness) and secondary (safety) outcomes. Abbreviations: AA: antiplatelet agents; CPABRS: commercial Chinese polyherbal preparation for activating blood and resolving stasis.

Adjusted logistic regression analyses (Figure 2) demonstrated that AA + CPABRS was associated with a significantly lower risk of MACCEs than AA monotherapy (OR 0.687, 95% CI, 0.544–0.876). CPABRS alone was associated with a numerically lower, but not statistically significant, risk of MACCEs (OR 0.820, 95% CI, 0.597–1.126). Additional Cox regression analyses and IPTW-weighted logistic regression analysis suggested similar results (Supplementary Appendix 2). These findings indicate that the adjunctive use of CPABRS with antiplatelet therapy may be associated with improved ischemic outcomes in older ICCD patients.

### Subgroup analyses

**FIGURE 3:**
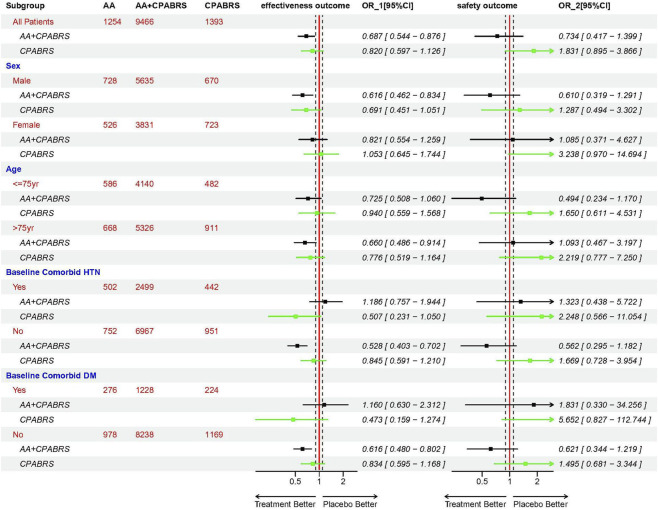
Subgroup analyses of the association between treatment group and effectiveness and safety outcomes. Abbreviations: AA: antiplatelet agents; CPABR: commercial Chinese polyherbal preparation for activating blood and resolving stasis; HTN: hypertension; DM: diabetes.

The subgroup analyses were generally consistent with the main analysis (Figure 3). AA + CPABRS use was associated with a lower risk of MACCEs in both sex subgroups (male and female) and in both age subgroups (≤75 years and >75 years). However, this association was not observed among patients with hypertension (OR, 1.186; 95% CI, 0.757–1.944) or diabetes mellitus (OR, 1.160; 95% CI, 0.630–2.312).

### Sensitivity analyses

**FIGURE 4:**
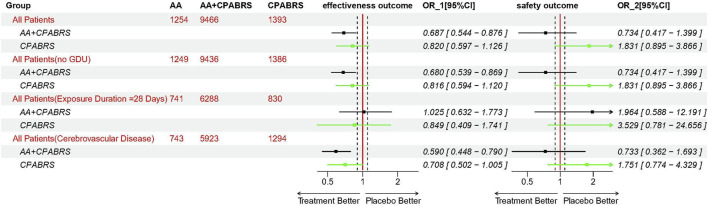
Sensitivity analyses of the association between treatment group and effectiveness and safety outcomes. Abbreviations: AA: antiplatelet agents; CPABRS: commercial Chinese polyherbal preparation for activating blood and resolving stasis.

The sensitivity analyses further supported the robustness of the primary findings (Figure 4). The association between AA + CPABRS use and lower MACCE risk remained significant after exclusion of patients with baseline gastroduodenal ulcers (OR, 0.680; 95% CI, 0.539–0.869) and after exclusion of those with ischemic cardiovascular disease (OR, 0.590; 95% CI, 0.448–0.790).

### Most frequently prescribed CPABRS products and MACCE risk

**FIGURE 5:**
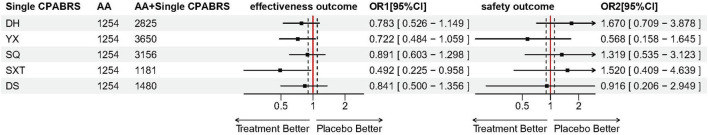
Association of the most frequently prescribed CPABRS products combined with AA with effectiveness and safety outcomes. Abbreviations: AA: antiplatelet agents; CPABRS: Chinese patent medicine for activating blood and resolving stasis; YX: Yinxing (Ginkgo biloba); DS, Danshen (Salvia miltiorrhiza Bunge); DS_SF, Danhong (Salvia miltiorrhiza and Carthamus tinctorius); SQ: Sanqi (Panax notoginseng); SZ_DL, Shuxuetong (Hirudo and Eisenia fetida).

The five most frequently prescribed CPABRS products in combination with AA were Yinxing (Ginkgo biloba), Danshen (Salvia miltiorrhiza Bunge), Danhong (Salvia miltiorrhiza; Carthamus tinctorius), Sanqi (Panax notoginseng), and Shuxuetong (Hirudo; *Eisenia fetida*) (Figure 5). For each of these formulations, the incidence of MACCEs was lower in the AA + CPABRS group than in the AA-alone group, although the magnitude of the difference varied across products. The overall pattern supported a beneficial association between AA + CPABRS and MACCEs.

### Secondary outcome: bleeding events

During follow-up, 105 patients (0.8%) experienced bleeding events. The incidence of bleeding events was 0.76% in the AA + CPABRS group, 1.44% in the CPABRS group, and 1.04% in the AA groups, respectively. In the adjusted analyses AA + CPABRS was not associated with a significantly different risk of bleeding events compared with AA alone (OR, 0.734; 95% CI, 0.417–1.399) (Figure 2).

## Discussion

To our knowledge, this population-based, real-world cohort study is the first to investigate the association of combined AA and CPABRS use with clinical outcomes in older patients with ICCD in China. Using a large medical claims database, we found that AA + CPABRS therapy was associated with a 31% lower risk of MACCEs compared with AA monotherapy, and this pattern remained consistent across multiple subgroup and sensitivity analyses. In addition, combination therapy was not associated with increased bleeding risk. These findings provide real-world evidence to suggest that CPABRS may have potential as an adjunctive option for secondary prevention in older adults with ICCD.

The management of ICCD requires balancing thrombotic and bleeding risks. International guidelines for ischemic cardiovascular diseases recommended bleeding-mitigation strategies (e.g., shortening dual antiplatelet therapy or de-escalating P2Y12 inhibitors), but similar guidance is lacking for cerebrovascular disease. Moreover, CPABRS have not been incorporated into international recommendations because robust supporting evidence remains insufficient. Chinese guidelines recognize CPABRS only for symptomatic benefits, such as angina relief or neurological recovery (Doctor Society of Integrative Medicine CMDA, 2018; Medicine CAoi et al., 2023), and provide no clear guidance on hard endpoints like MACCEs or their role in balancing ischemic and bleeding risks.

Only a few CPABRS products [e.g., tongxinluo (Yang et al., 2023), xinyue capsule (Guo et al., 2020), dengzhanshengmai capsule (Cai et al., 2019), and ginkgolide (Dong et al., 2021)], have been studied with antiplatelet therapy for ischemic risk reduction, but these trials focused on younger or general adults. Evidence in older patients (≥65 years) remains scarce, who have distinct pathophysiological features such as impaired endothelial function, altered drug metabolism, increased inflammation, polypharmacy, and multimorbidity (Capodanno and Angiolillo, 2010; Katsimpris et al., 2019; Nicholson et al., 2024; Toda, 2012). Moreover, prior studies had small sample sizes and lacked large-scale medical big data. Our population-based cohort study extends the evidence by showing that, among older ICCD patients, adding CPABRS to antiplatelet therapy was associated with better clinical outcomes. Notably, this benefit was observed even in patients aged ≥75 years, who comprised over half of our study population.

The observed association may be biologically plausible, as CPABRS may confer cardiovascular and cerebrovascular protection through multiple pathways, including antiplatelet, anti-inflammatory, antioxidant, and microcirculatory effects. In our study, the five most frequently prescribed CPABRS (Yinxing (Ginkgo biloba), Danshen (Salvia miltiorrhiza), Danhong (Salvia miltiorrhiza and Carthamus tinctorius), Sanqi (Panax notoginseng), and Shuxuetong (Hirudo and *Eisenia fetida*)) have documented pharmacological activities that may complement AA therapy. For instance, Ginkgo biloba inhibits platelet aggregation by reducing arachidonic acid and modulating platelet-activating factor pathways (Cho and Nam, 2007; Ke et al., 2021). Salvia miltiorrhiza improves microcirculation by downregulating endothelin-1, iNOS, VEGF, P-selectin, and inflammatory cytokines) (Park et al., 2008; Sun et al., 2020). These pharmacological properties may help explain why adding CPABRS to antiplatelet therapy was associated with a lower risk of MACCEs without an apparent increase in bleeding risk.

The age-specific patterns observed in this study warrant further investigation. Adding CPABRS to antiplatelet therapy showed a protective trend in both younger (≤75) and older (>75) patients, but bleeding risk differed between groups. The >75 group may be more vulnerable to bleeding due to endothelial dysfunction, comorbidities, and polypharmacy (Katsimpris et al., 2019; Nicholson et al., 2024). In addition, elevated inflammatory markers (e.g., IL-6, CRP), more common in older adults, may complicate the balance between ischemic and bleeding risks (Bochaton et al., 2022; Mousavi et al., 2022).

In subgroup analyses, no protective association was observed for AA + CPABRS in patients with hypertension or diabetes. This may be due to their higher baseline vascular risk and more intensive background treatment, which could attenuate the incremental benefit of adjunctive therapy (Petrie et al., 2018; Brandes, 2014). In addition, key variables like blood pressure and glycemic control were unavailable in the claims database, while poor control of blood pressure and blood glucose are associated with vascular injury and cardiovascular risk. Moreover, the subgroup sample sizes were relatively small with wide confidence intervals, limiting statistical precision. Therefore, these findings should be interpreted as exploratory and hypothesis-generating rather than as definitive evidence that CPABRS offers no benefit in patients with hypertension or diabetes.

This study has several strengths. First, it adds population-based real-world evidence from a large claims database, helping address the limited sample sizes of previous randomized controlled trials and observational studies. Second, it focuses on older patients, an understudied but clinically important population, including those aged ≥75 years. Third, the analytical strategy adjusted for multiple clinically relevant confounders and incorporated extensive subgroup, sensitivity, additional Cox regression, and IPTW-weighted logistic regression analyses, thereby strengthening the robustness of the findings.

However, this study has several limitations. First, the study population was restricted to insured residents of Beijing, which may limit the generalizability of the findings. Second, despite multiple covariates were adjusted for, residual confounding may exist due to unavailable data on lifestyle factors, disease severity, laboratory parameters, and imaging findings. Third, only short-term outcomes were assessed, and the long-term associations remain to be determined. However, observing a signal even within a brief follow-up suggests clinical relevance. In addition, published studies (Cao et al., 2020; Westerhout et al., 2013) suggest that, in short-term analyses, the absence of death data is unlikely to alter the overall pattern of associations. Moreover, data were only available from 2016 to 2017(restrictions in the BMMID), but the treatment combination (AA + CPABRS) remains routine in clinical practice, and the therapeutic rationale has not fundamentally changed. Thus, these data still provide useful evidence on short-term safety and outcomes in older ICCD patients. Furthermore, CPABRS were analyzed as a single therapeutic class because they share the common therapeutic principle of activating blood and resolving stasis and are often used under a similar therapeutic framework in practice. Therefore, findings should be interpreted at the class level, not as evidence for individual formulations. Further studies focusing on specific CPABRS products are needed to define formulation-specific effectiveness and safety. Finally, although the five most used CPABRS products were associated with favorable outcomes, the sample sizes were insufficient for a rigorous evaluation of individual formulations, and future studies targeting each CPABRS are needed.

## Conclusion

Among older patients with ICCD, adjunctive use of CPABRS with antiplatelet therapy was associated with a lower risk of MACCEs compared with antiplatelet therapy alone, without a statistically significant increase in bleeding events. These findings suggest that AA + CPABRS may be associated with potential short-term clinical benefit in this high-risk population, while maintaining acceptable safety. Future studies should explore the long-term outcomes, mechanisms of action, and comparative effectiveness of specific CPABRS formulations to optimize therapeutic strategies for elderly patients with ICCD.

## Data Availability

The data analyzed in this study were obtained from the Beijing Municipal Medical Insurance Database under authorization for the present research. Because the dataset contains sensitive personal health and insurance information, it cannot be deposited in a public repository due to legal, ethical, and privacy restrictions. Therefore, no public repository accession number is available.
