# Supplementary material for: Association of blood-activating commercial Chinese polyherbal preparation with clinical outcomes in older patients with ischemic cardiovascular or cerebrovascular diseases: a real-world cohort study
Source: Front Pharmacol. 2026 Jun 17;17:1819226. doi: 10.3389/fphar.2026.1819226 (PMC13319001; doi:10.3389/fphar.2026.1819226)
Supplement: Supplementary file 1 [file Table2.docx]

# Cox proportional hazards regression

In the additional Cox regression analyses, AA + CPABRS remained significantly associated with a lower risk of MACCEs compared with AA alone (HR = 0.642, 95% CI 0.510–0.808, *P* < 0.001), whereas CPABRS alone was not significantly associated with MACCE risk (HR = 0.795, *P* = 0.140). For bleeding outcomes, neither AA + CPABRS (HR = 0.668, *P* = 0.183) nor CPABRS alone (HR = 1.565, *P* = 0.222) showed a statistically significant association. These results were consistent with the primary logistic regression analyses.(Figure 1-4)


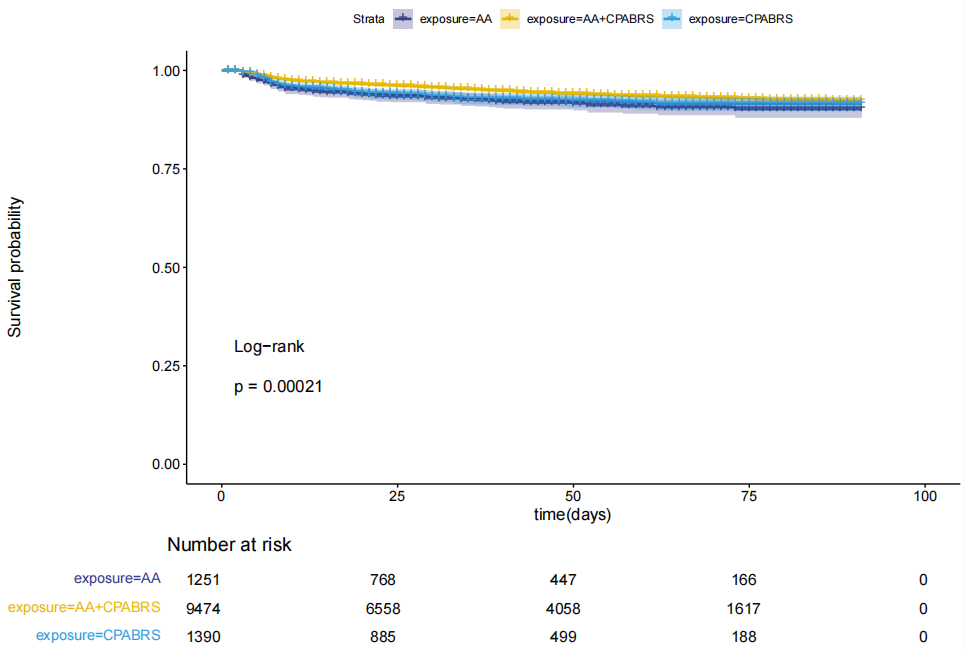


**Figure 1 Kaplan–Meier curves for time to first MACCEs**

AA: antiplatelet agents; CPABRS: Commercial Chinese polyherbal preparation for activating blood and resolving stasis.


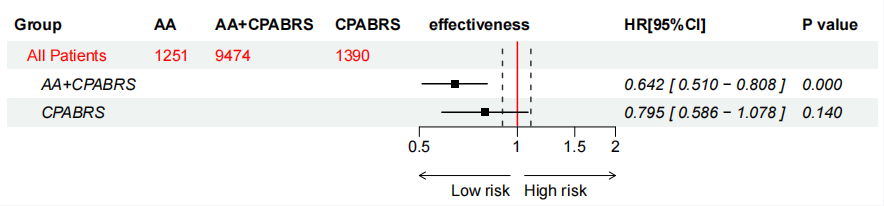


**Figure 2 Hazard ratio for MACCEs**

AA: antiplatelet agents; CPABRS: Commercial Chinese polyherbal preparation for activating blood and resolving stasis.


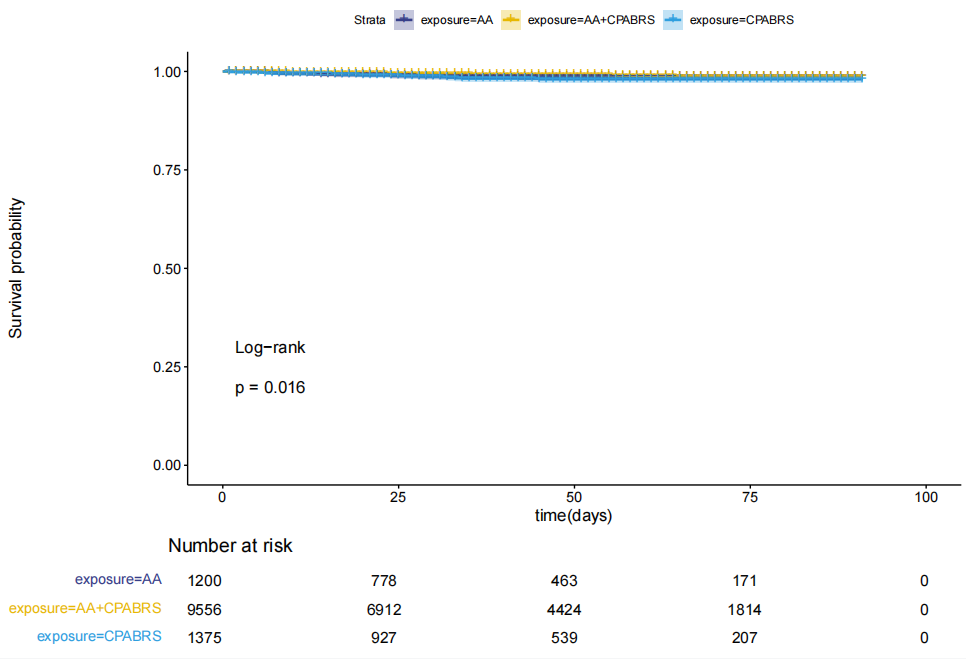


**Figure 3 Kaplan–Meier curves for time to first bleeding events**

AA: antiplatelet agents; CPABRS: Commercial Chinese polyherbal preparation for activating blood and resolving stasis.


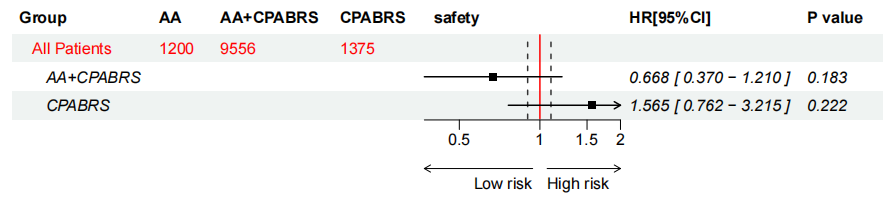


**Figure 4 Hazard ratio for bleeding events**

AA: antiplatelet agents; CPABRS: Commercial Chinese polyherbal preparation for activating blood and resolving stasis.

# Inverse probability of treatment weighting (IPTW) based on propensity scores

Table 1 presents the baseline characteristics of the three groups after IPTW. After IPTW, the AA + CPABRS group remained significantly associated with a lower risk of the MACCEs (OR, 0.636; 95% CI, 0.498–0.813; P < 0.001), consistent with the pre-weighting analysis (OR 0.687, 95% CI, 0.544–0.876). CPABRS alone was associated with a numerically lower, but not statistically significant, risk of MACCEs (OR 0.807, 95% CI, 0.569–1.146), consistent with the unweighting analysis (OR 0.820, 95% CI, 0.597–1.126). (Figure 5).

For bleeding events, neither AA + CPABRS (OR, 0.650; 95% CI, 0.349-1.210) nor CPABRS alone (OR, 1.332, 95% CI, 0.607-2.924) showed a statistically significant association. These results were consistent with the unweighting logistic regression analysis. (Figure 6)

**Table 1 Patient Demographic and Baseline Characteristics after IPTW**

|  | AA  (n=1251.9) | AA+CPABRS  (n=9469.7) | CPABRS  (n=1386.6) | P value |
| --- | --- | --- | --- | --- |
| sex = female (%) | 518.2 (41.4) | 3976.1 (42.0) | 601.0 (43.3) | 0.625 |
| age (mean (SD)) | 76.55 (6.85) | 76.53 (6.83) | 76.63 (7.09) | 0.923 |
| Anticoagulants = yes (%) | 497.6 (39.7) | 3818.2 (40.3) | 562.2 (40.5) | 0.920 |
| Antidiabetic = yes (%) | 578.9 (46.2) | 4286.8 (45.3) | 631.3 (45.5) | 0.844 |
| Hypolipidemic = yes (%) | 1061.0 (84.8) | 7987.3 (84.3) | 1169.4 (84.3) | 0.913 |
| Antihypertensive = yes (%) | 1051.7 (84.0) | 7957.7 (84.0) | 1164.0 (83.9) | 0.997 |
| AD_AN = yes (%) | 2.5 (0.2) | 16.4 (0.2) | 0.0 (0.0) | 0.226 |
| CKD = yes (%) | 26.7 (2.1) | 178.6 (1.9) | 30.0 (2.2) | 0.707 |
| GDU = yes (%) | 4.6 (0.4) | 35.3 (0.4) | 7.3 (0.5) | 0.720 |
| CVD = yes (%) | 30.3 (2.4) | 213.3 (2.3) | 41.9 (3.0) | 0.444 |
| DM = yes (%) | 188.7 (15.1) | 1350.4 (14.3) | 199.9 (14.4) | 0.753 |
| ED = yes (%) | 3.5 (0.3) | 31.7 (0.3) | 4.7 (0.3) | 0.919 |
| HLP = yes (%) | 165.0 (13.2) | 1222.2 (12.9) | 185.0 (13.3) | 0.896 |
| HTN = yes (%) | 362.3 (28.9) | 2688.9 (28.4) | 393.0 (28.3) | 0.925 |

Note: IPTW generates a weighted pseudo-population, which can result in non-integer values for variables that are originally expressed as whole numbers. Therefore, the numbers of patients in the three exposure groups are not presented in the subsequent forest plots. AA: antiplatelet agents; CPABRS: Commercial Chinese polyherbal preparation for activating blood and resolving stasis.


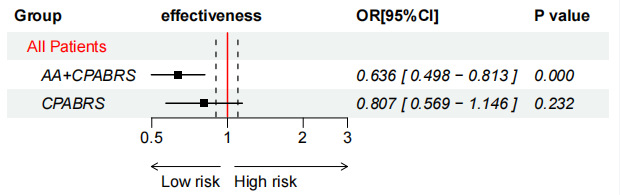


**Figure 5 Odds ratio after IPTW for MACCEs**

AA: antiplatelet agents; CPABRS: Commercial Chinese polyherbal preparation for activating blood and resolving stasis.


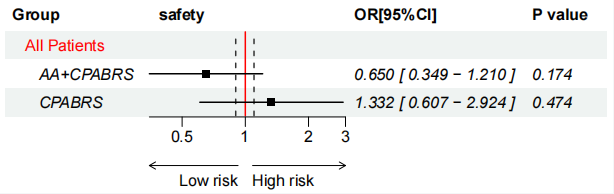


**Figure 6 Odds ratio after IPTW for bleeding events**

AA: antiplatelet agents; CPABRS: Commercial Chinese polyherbal preparation for activating blood and resolving stasis.
